# Leukocyte Telomere Length in the Finnish Diabetes Prevention Study

**DOI:** 10.1371/journal.pone.0034948

**Published:** 2012-04-06

**Authors:** Iiris Hovatta, Vanessa D. F. de Mello, Laura Kananen, Jaana Lindström, Johan G. Eriksson, Pirjo Ilanne-Parikka, Sirkka Keinänen-Kiukaanniemi, Markku Peltonen, Jaakko Tuomilehto, Matti Uusitupa

**Affiliations:** 1 Research Programs Unit, Molecular Neurology, Biomedicum-Helsinki, University of Helsinki, Helsinki, Finland; 2 Department of Medical Genetics, Haartman Institute, University of Helsinki, Helsinki, Finland; 3 Mental Health and Substance Abuse Services, National Institute for Health and Welfare, Helsinki, Finland; 4 Institute of Public Health and Clinical Nutrition, Clinical Nutrition, University of Eastern Finland, Kuopio, Finland; 5 Department of Health Promotion and Chronic Disease Prevention, National Institute for Health and Welfare, Helsinki, Finland; 6 Department of General Practice and Primary Health Care, University of Helsinki, Helsinki, Finland; 7 Folkhälsan Research Center, Helsinki, Finland; 8 Unit of General Practice, Helsinki University Central Hospital, Helsinki, Finland; 9 Vaasa Central Hospital, Vaasa, Finland; 10 Diabetes Centre, Finnish Diabetes Association, Tampere, Finland; 11 Science Centre, Pirkanmaa Hospital District, Tampere University Hospital, Tampere, Finland; 12 Institute of Health Sciences, University of Oulu, Oulu, Finland; 13 Unit of General Practice, Oulu University Hospital and Oulu Health Centre, Oulu, Finland; 14 Diabetes Prevention Unit, National Institute for Health and Welfare, Helsinki, Finland; 15 South Ostrobothnia Central Hospital, Seinäjoki, Finland; 16 Center for Vascular Prevention, Danube-University Krems, Krems, Austria; 17 Research Unit, Kuopio University Hospital, Kuopio, Finland; Innsbruck Medical University, Austria

## Abstract

Leukocyte telomere length (TL) is considered a biomarker for biological aging. Shortened TL has been observed in many complex diseases, including type 2 diabetes (T2DM). Lifestyle intervention studies, e.g. the Diabetes Prevention Study (DPS), have shown a decrease in the incidence of T2DM by promoting healthy lifestyles in individuals with impaired glucose tolerance (IGT). Our aim was to study in the DPS the influence of the lifestyle intervention on TL. TL was measured by quantitative PCR-based method at two time points (N = 334 and 343) on average 4.5 years apart during the active intervention and post-intervention follow-up. TL inversely correlated with age. Our main finding was that TL increased in about two thirds of the individuals both in the intervention and in the control groups during follow-up; TL increased most in individuals with the shortest TL at the first measurement. TL was not associated with development of T2DM, nor did lifestyle intervention have an effect on TL. No association between insulin secretion or insulin resistance indices and TL was observed. We did not detect an association between TL and development of T2DM in the DPS participants. It could be due to all participants being overweight and having IGT at baseline, both of which have been found to be independently associated with shorter leukocyte TL in some earlier studies. TL had no substantial role in worsening of glucose tolerance in people with IGT. Our study confirms that leukocyte TL can increase with time even in obese people with impaired glucose metabolism.

## Introduction

Both genetic and environmental factors contribute to the development of type 2 diabetes (T2DM) [1]. It has been shown that obese individuals with impaired glucose metabolism, who are at increased risk of developing T2DM, can decrease their risk of developing T2DM by lifestyle changes involving a healthy diet, increased physical activity and moderate weight loss [2].

Telomeres are the regions at the ends of the chromosomes and consist of DNA repeat sequence and associated proteins. It has been suggested that leukocyte telomere length (TL) is a biomarker of biological aging, which can be used to predict morbidity and mortality [3]. Leukocytes are the most intensively studied cell type due to their easy access, and TL dynamics in other tissues are not characterized in similar detail. In humans, the strongest evidence for leukocyte TL as a predictor of mortality comes from twin studies [4], [5]. In somatic cells, TL shortens at cell division, while in stem cells TL is maintained by the telomerase enzyme [6]. It has recently been understood that while overall TL shortens by age, at the individual level TL is a dynamic feature, and TL can also increase in leukocytes over time [7]–[9].

TL is a complex phenotype affected by genes [10]–[13] and environmental factors, such as smoking [14] and physical activity [15]. Accelerated telomere shortening occurs in certain monogenic diseases associated with premature aging, including ataxia telangiectasia, Werner’s syndrome, and Bloom’s syndrome [6]. In addition, shortened TL has been observed in a large number of complex diseases, with replicated findings in cardiovascular diseases [16], [17]. This implies that chronic age-related diseases may be associated with aging process per se.

T2DM is known to shorten life expectancy compared with the general population, mostly due to cardiovascular diseases [18], [19]. T2DM has been recently shown to associate with shortened TL in leukocytes [20]–[24] and monocytes [25]. Risk factors for T2DM have also been associated with shorter TL in some studies [26], [27]. In addition, telomerase deficiency has been shown to impair glucose metabolism and insulin secretion [28].

It has been suggested that lifestyle changes, including a healthier diet, moderate aerobic exercise, and stress management can increase leukocyte telomerase enzyme activity [29]. However, it is not known whether lifestyle changes affect leukocyte TL. We set to investigate whether lifestyle changes in the DPS including weight loss, increased physical activity and healthy diet affect leukocyte TL. In addition, we studied telomere dynamics over the follow-up period, and investigated the relationship between TL and insulin secretion and resistance, and risk of developing T2DM.

## Methods

### Ethic Statement

The study protocol was approved by the Ethics Committee of the National Public Health Institute (currently National Institute for Health and Welfare) of Helsinki, Finland, and all participants gave written informed consent.

### Study Sample

#### Design of DPS

The DPS was a randomized, controlled, multicenter study carried out in Finland between the years 1993 and 2000 (ClinicalTrials.gov NCT00518167). A total of 522 individuals with impaired glucose tolerance (IGT) were randomized into either an intervention or control group in five centers starting between the years 1993–1998. The study design and methods of the DPS have been reported in detail elsewhere [30], [31]. The main inclusion criteria were as follows: BMI >25 kg/m^2^, age 40–64 years, and IGT based on the mean values of two OGTTs based on the World Health Organization 1985 criteria. The median length of the original randomized intervention study was 4 years (range 1–6 years).

#### Program for the intervention group

The intervention program has been described previously in detail [30], [31]. Briefly, the individuals in the intervention group were given individually tailored dietary advice aiming at reducing weight and the intake of total and saturated fat and increasing the intake of dietary fiber. In addition, individuals in the intervention group also received individual guidance to increase their level of physical activity. The control group received general advice on the benefits of weight reduction, physical activity and a healthy diet.

### Glucose and Insulin Measurements

Glucose levels were measured locally by standard methods, and the measurements were standardized by the central laboratory in Helsinki [31]. Serum insulin was determined with a radioimmunoassay in the central laboratory in Helsinki (Pharmacia, Uppsala, Sweden). To estimate insulin secretion and insulin sensitivity, we calculated the ratio of total insulin area under the curve (AUC) and total glucose AUC during the 0–30 min OGTT (AIGR_0–30_) and the Matsuda index of insulin sensitivity (Matsuda ISI: 10,000/square root of [fasting glucose x fasting insulin × [arithmetic mean of glucose × arithmetic mean insulin during an OGTT]]) using glucose and insulin concentrations from samples taken during an OGTT at 0, 30, and 120 min. These data were available for the visits between the years 1 and 4 of the active randomized study period. Based on the known nonlinear relationship between insulin secretion and insulin sensitivity we also adjusted, by regression analysis, the log of AIGR_0–30_ by the log of the Matsuda ISI at each year of the study to obtain insulin secretion independently of insulin sensitivity.

### TL Measurement

Sampling for DNA extraction aiming at TL studies were taken at two occasions. The first sampling was taken between the years 1997 and 1998 (ranging from baseline (n = 27) to 4 years in the study trial; mean of 1.2 years), and the second sampling, between the years 2001 and 2002 (ranging from 1 to 7 years of follow-up after the study had ended; mean of 1.5 years). DNA samples at two time points were available from 378 individuals comprising of 380 intervention cases and 376 controls (498 females and 258 males).

DNA was extracted from peripheral blood leukocytes by salt precipitation method. The relative telomere length was determined from DNA by a quantitative real-time PCR-based method [32], [33] with the following modifications. ß-hemoglobin was used as a single copy reference gene. PCR reactions were performed separately for telomere and ß-hemoglobin in paired 384 well plates in which matched sample well positions were used. A genomic DNA dilution series (0.5, 1.0, 2.0, 5.0, 10, 20 and 30 ng) was included in every plate to create a standard curve, which was used to perform absolute quantification of each DNA sample. Ten ng of DNA was used for each individual reaction. Samples were randomized according to sex and intervention group with an equal number on each plate. The first and the second DNA sample of each individual were placed on the same qPCR plate. Samples and standard dilutions were transferred into 384-well plates as triplicates using DNA Hydra 96 robot (Art Robbins Instruments, Sunnyvale, VA, USA) and dried for 24 h at +37°C. ß-hemoglobin qPCR mix included 300 nM Hgb1 primer (5′-GCTTCTGACACAACTGTGTTCACTAGC-3′) and Hgb2 primer (5′-CACCAACTTCATCCACGTTCACC-3′) in a total volume of 15 µl of iQ SyBrGreen supermix (Bio-Rad). The reaction conditions were 95°C for 3 min followed by 35 cycles at 95°C for 15 s, 58°C for 20 s and 72°C for 20 s. Telomere qPCR mix content was 270 nM tel1b primer (5′-CGGTTT(GTTTGG)5GTT-3′) and 900 nM tel2b primer (5′-GGCTTG(CCTTAC)5CCT-3′), 1% DMSO (Sigma), 0.2 mM of each dNTP (Fermentas), 1.5 mM MgCl_2_ (Applied Biosystems International), 5 mM DTT (Sigma), 0.2X SYBR Green I (Invitrogen), and 1.25 U AmpliTaq Gold DNA polymerase (Applied Biosystems International) in a total volume of 15 µl AmpliTaq Gold Buffer II. The cycling conditions were 10 min at 95°C followed by 25 cycles at 95°C for 15 s and 54°C for 2 min. Both reactions were performed with CFX 384 Real-Time PCR Detection System (Bio-Rad, Hercules, CA, USA). Specific primer binding was controlled by Melt-curve analysis.

To perform quality control we used the Bio-Rad CFX Manager software v.1.6. At this point triplicates with amplification curve standard deviation above 0.5 at the threshold level were omitted (telomere N = 17; ß-hemoglobin N = 26). The number of wells without any amplification was 23 for telomere reaction and four for ß-hemoglobin reaction. The average correlation coefficient of the standard curves was 0.997 for both the telomere and the ß-hemoglobin reaction. The corresponding average PCR efficiencies were 92.1% and 83.2%, respectively. All plates included five genomic DNA control samples for the plate effect calibration and for monitoring repeat measures correlation coefficient of variation (CV). The control sample group means in every plate were calculated for the calibrator values and these were used to adjust the qPCR plate effect (ratio separately from telomere or ß-hemoglobin reaction and specific calibrator). The T/S (telomere to single copy gene intensity) ratios from calibrated values were calculated to obtain the relative TL for the samples. The quantities of the control samples were used for obtaining the CV values as the ratio of the standard deviation to the mean, and those were on average 7.0% for the telomere reaction, 7.5% for the ß-hemoglobin reaction, and 14.2% for their ratio (T/S).

### Statistical Analysis

Statistical analyses were performed with SPSS software version 18.0 (SPSS Inc., Chicago, IL) and visualized with SigmaPlot version 9.0. If TL deviated over 3 standard deviations from the mean, the sample was omitted as an outlier (N = 14). The final sample consisted of 677 samples with TL measurement (311 individuals with both time points). The relationship between the study groups and TL was examined by Univariate GLM adjusted for age, sex and also adjusted for TL at the first DNA sampling when the change in TL over time was tested. The association of TL with the risk of incident T2DM during a mean of 8.5 years of follow-up after the first TL measurement, and after a mean of 5.5 years of follow-up after the second DNA sampling was assessed by Cox proportional hazards regression models adjusted for age, sex and randomization group, and also adjusted for TL at the first DNA sampling when the change in TL over time was tested. For this purpose, individuals already diagnosed with T2DM according to WHO criteria [31], [34] at the beginning of each follow-up were excluded from the analyses. The results are described as the respective hazard ratios (HR) for the risk of developing T2DM during each of the respective follow-up periods according to a 1-SD change in TL. The proportional hazard assumption was evaluated and confirmed using the Schoenfeld residual which was plotted against time for each of the models. A *P* value of <0.05 was considered statistically significant.

## Results

### Correlation of TL with Age and Sex

**Figure 1:**
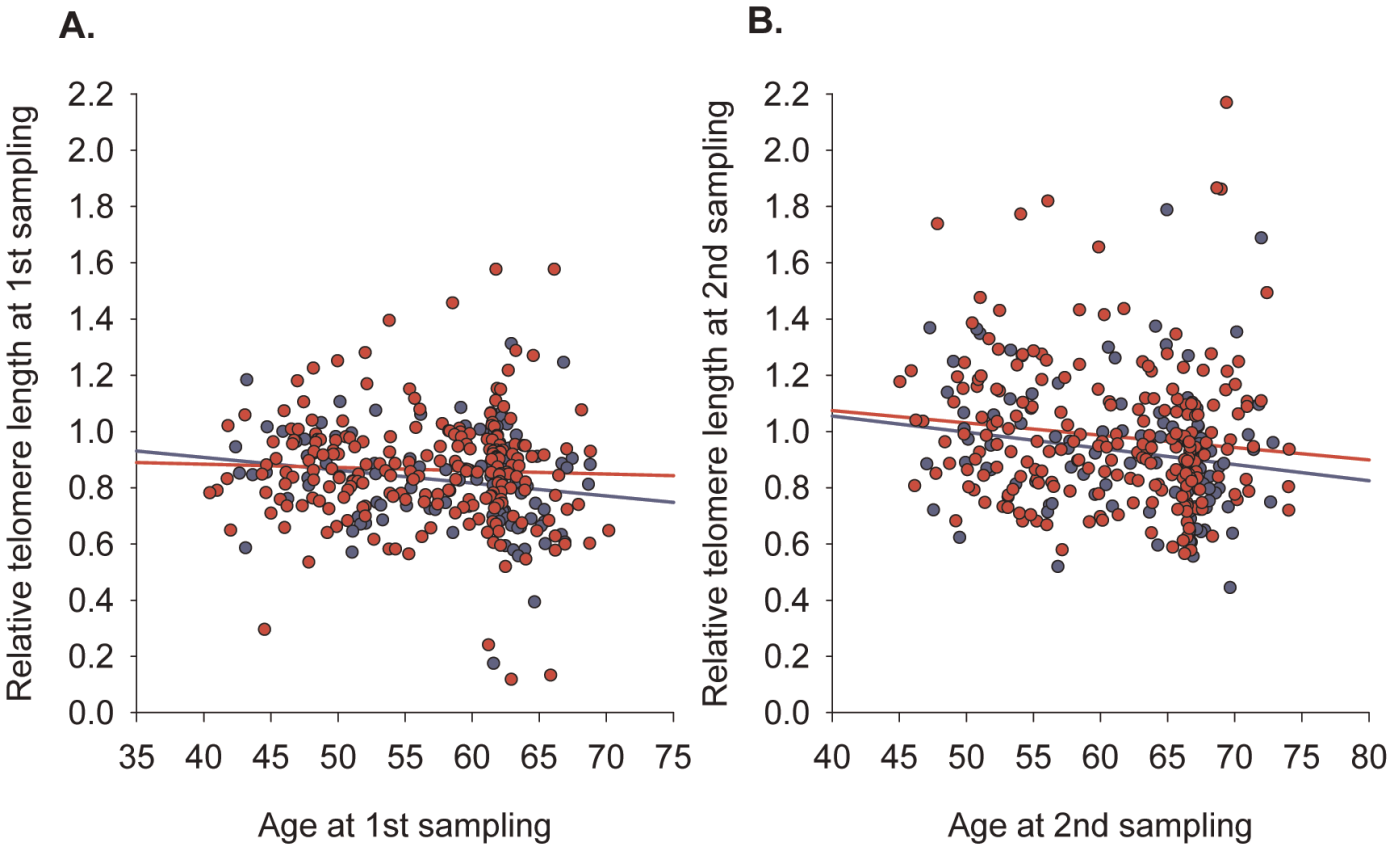
Telomere length as a function of age. Females are shown with red dots and males with blue dots, each dot representing one individual. Regression lines for both groups are shown with the same color coding. A) the first DNA sample; B) the second DNA sample.

As expected, relative TL correlated inversely with age at both first and second DNA sampling (β = −0.11, p = 0.06 and β = −0.14, p = 0.04, respectively; Figure 1). In the entire sample, women had longer relative TL than men at both first (0.86 ± 0.19 vs. 0.83 ± 0.17, p = 0.09) and second (0.98 ± 0.25 vs. 0.93 ± 0.23, P = 0.09) DNA sampling, but the finding did not reach statistical significance even when adjusting for age and randomization group (p = 0.14 and p = 0.08, respectively; Figure 1).

### TL According to Randomization Group

There was no significant difference in the relative TL between the intervention and control groups at the time of the first (p = 0.31) or second sampling (p = 0.76) (Table 1). Adjustments for the time (years) in the study or in the follow-up at sampling did not change the results. Results were similar when participants with T2DM during the randomized trial period or during the entire follow-up were excluded (p = 0.36 and p = 0.86).

**Table 1:** **The Diabetes Prevention Study sample.**

Characteristic	Intervention cases	Controls
**Number of individuals (n)**	190	188
1st DNA sample available (n)	162	172
2nd DNA sample available (n)	171	172
**Women**	64.7%	67.0%
**Age at inclusion to the study (years)**	55.83 (±7.17)	55.45 (±6.97)
**Age at DNA sampling (years)**		
1st DNA sample	57.07 (±7.06)	56.69 (±6.98)
2nd DNA sample	61.58 (±7.09)	61.17 (±6.97)
**Time between 1st and 2nd DNA sample (years)**	4.56 (±0.57)	4.53 (±0.68)
**Relative telomere length**		
1st DNA sample	0.84 (±0.18)	0.86 (±0.18)
2nd DNA sample	0.96 (±0.23)	0.97 (±0.26)
**Telomere length yearly change, n**	0.027 (±0.052), 150	0.022 (±0.048), 161
**BMI (kg/m^2^)**		
1st DNA sample	29.8 (±4.2)	30.8 (±4.7)
2nd DNA sample	30.4 (±4.9)	31.2 (±5.1)
Change (between 1st and 2nd DNA sampling)	0.49 (−6.10; 8.77)	0.46 (−5.57; 9.06)

Data are number of individuals, mean ± SD, median (range) or %.

### TL Change Over Time

**Figure 2:**
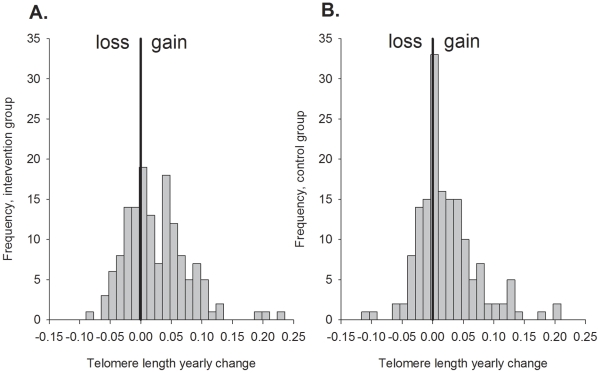
Telomere length change between the 1^st^ and 2^nd^ DNA sampling. Diabetes Prevention Study sample A) intervention cases and B) controls as a histogram with frequencies of telomere length difference between two time points.

**Figure 3:**
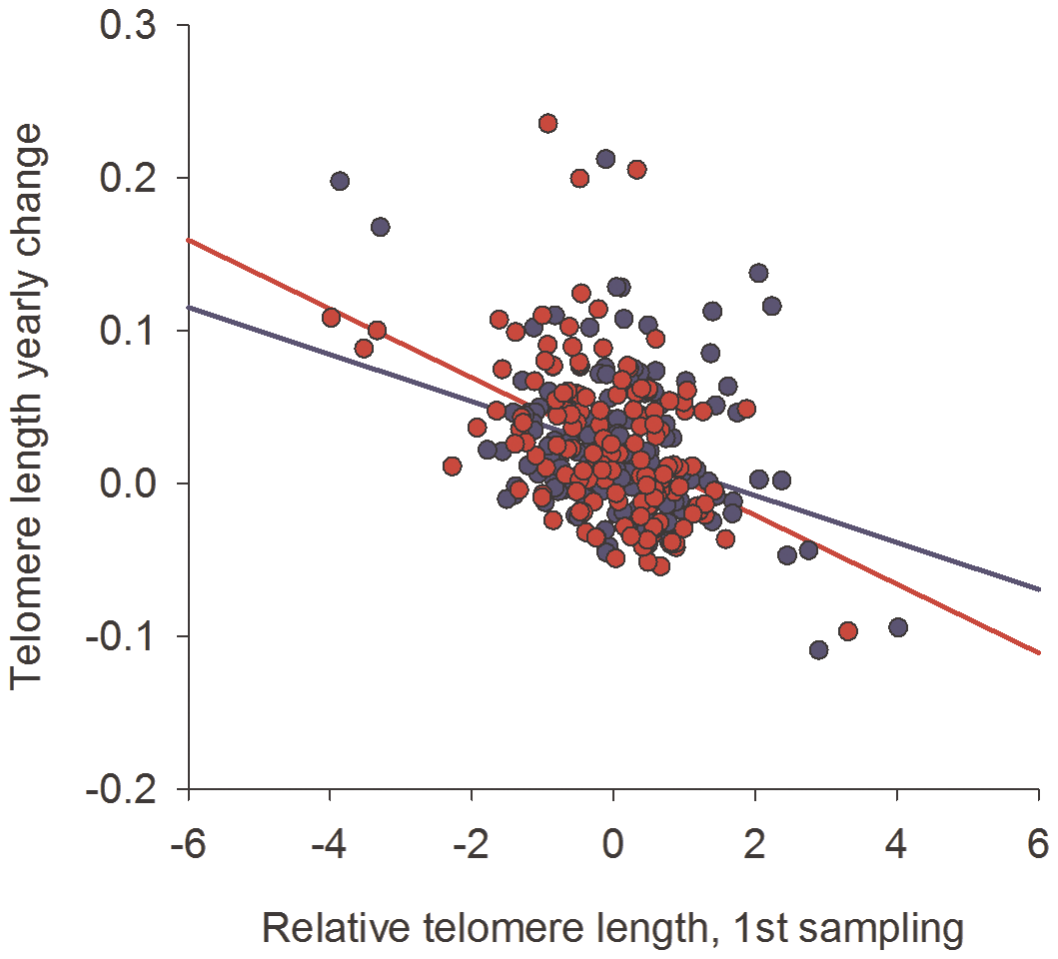
Telomere length yearly change as a function of telomere length at the 1^st^ DNA sampling. Diabetes Prevention Study sample intervention cases (N = 190) are shown with red dots and controls (N = 188) with blue dots, each dot representing one individual. Regression lines for both groups are shown with the same color coding. Relative telomere length is adjusted for age and sex.

The mean time period between the first and second sampling was 4.5 years (Table 1). We calculated the yearly change in TL as the TL at the second sampling minus the TL at the first sampling divided by the time between the samplings. Surprisingly, relative TL during this about 4.5 year period increased in about two thirds of the individuals (Figure 2). The yearly change in TL depended on the TL at the first measurement (r = −0.39, p<0.001; Figure 3); the individuals who had the shortest TL at the first measurement showed the largest increase in TL. There was no difference in the TL change rate between the intervention and control groups (p = 0.65; Table 1). Exclusion of individuals diagnosed with T2DM during the randomized trial phase (first DNA sampling) did not change the results (p = 0.49).

### TL and BMI

Considering the individuals who had TL measured at both DNA sampling, there was a slight increase in BMI between the measurements within the average of 4.5 years (mean ± SD: 30.4 ± 4.5 vs. 30.9 ± 4.9 kg/m^2^), which was not statistically different between the study groups (p = 0.11, Table 1). In simple correlation analyses, we did not find any correlation between BMI and the relative TL at first or second DNA sampling (r = 0.01, p = 0.83 and r = 0.03, p = 0.55, respectively). However, there was an inverse but weak correlation between yearly change in TL and change in BMI between the first and second DNA sampling (r = −0.12, p = 0.03), but after adjusting for age at sampling, sex, study group, and TL at first DNA sampling using multiple linear regression model the association was slightly attenuated (*β* = −0.11, p = 0.06).

### TL and T2DM

Over a mean of 8.5 years of follow-up (range 0.5 to 12.7 ) after the first DNA sampling, 130 of the 302 participants developed T2DM (5.1 per 100 person-years). Considering only individuals whose second DNA sample was also available, during these 8.5 years, 118 of 282 participants developed T2DM (5.0 per 100 person-years). In participants whose TL was measured only at the second DNA sampling, over a mean of 5.5 follow-up years (range 0.2 to 8.2 years) after the second DNA sampling has been taken, 66 of 245 participants developed T2DM (4.9 per 100 person-years).

In Cox regression models, we observed an inverse association between the increase in TL over the 4.5 years and T2DM development during the 8.5-year follow-up, although it was of borderline significance (HR: 0.81 [0.65; 1.00]; p = 0.05). The TL at the time of the first sampling and second DNA sampling, however, were not significantly inversely associated with the risk of developing overt T2DM during the 8.5 and 5.5 follow-up years, respectively (HR: 0.46 [0.18; 1.16]; p = 0.10 and (HR: 0.76 [0.49; 1.18]; p = 0.22, respectively).

We also tested if TL was related to Matsuda insulin sensitivity index (ISI) or to Matsuda ISI-adjusted AIGR_0–30_ as an estimate of β-cell function, as both indices have been shown to be associated with the risk of developing T2DM among the DPS participants [35]. The positive correlation between the yearly change in TL (r = 0.10) or TL at the first (r = 0.03) or second (r = 0.10) DNA sampling were not significantly associated with the average Matsuda ISI (4.03 ± 1.93) during the four-year study follow-up (p = 0.15, p = 0.57 and p = 0.09, respectively). We did not find any correlation between the Matsuda ISI-adjusted AIGR_0–30_ during the four-year study follow-up (mean ± SD: 30.3 ± 10.1) and TL at first or second sampling or the TL yearly change (r<0.05 and p>0.40 for all).

## Discussion

The association between TL and chronic diseases has mostly been studied in cross-sectional settings. In some studies published so far, a shorter leukocyte TL has been associated with T2DM or its risk factors [20]–[28]. T2DM can be prevented by changes in lifestyle in high-risk individuals as shown in the DPS [30], [31]. Therefore, in this study, we set out to investigate whether preventative measures affect leukocyte TL within the average follow-up of 4.5 years. Participants were middle-aged overweight people with IGT. They were randomized to either a usual care control group or an intensive lifestyle intervention group.

As expected, age was inversely correlated with TL. Interestingly, we detected an increase in TL in about two thirds of the DPS participants both in the intervention and in the control group. Individuals who had the shortest TL in the first sampling showed the greatest increase in TL. Increased leukocyte TL over time has been observed recently in a few studies [7]–[9]. In all of them, telomere attrition rate has depended on the initial TL both at a 10-year [7], [8] and 6-month [9] follow-up periods. Therefore, it seems that individual leukocyte TL is a dynamic feature and that TL can vary to both directions during a lifetime [9].

Our main finding was an increase in the TL over the 4.5 year follow-up period both in the intervention and in the control group. This is most likely due to both groups receiving at least some information regarding healthy lifestyle, although only the intervention group was counseled and monitored intensively. Our results suggest that TL may be linked to the progression from IGT to T2DM. However, although the lifestyle intervention resulted in a dramatic reduction in the risk of T2DM, it did not have any differential effect on TL between the two groups. Again, since the control group also received general information on a healthy lifestyle, this may have diminished putative differences between the groups. Telomere shortening is counteracted by the telomerase enzyme. Ornish et al. [29] recently showed in men with benign prostate cancer that a three-month intensive lifestyle change, consisting of healthier diet, moderate aerobic exercise, stress management, and group support sessions, increased telomerase activity in peripheral blood mononuclear cells. Our study and theirs are not easily comparable as TL was not measured by Ornish et al. and telomerase activity was not measured in our study.

In light of the fact that all participants had an increased risk for T2DM, i.e. were overweight and had IGT, it is not surprising that we did not see a stronger association with TL and T2DM diagnosis. Obesity and IGT have themselves been associated with shortened TL [14], [28], [36], [37]. Another limitation in our study is the lack of a population-based control group without any intervention. Such controls would have enabled us to study in more detail the effect of the intervention and the effect of development of T2DM on TL. Also, despite that both DNA samples of an individual were analyzed on the same qPCR plate for reliable measurement of TL yearly change, we cannot rule out the effect of DNA storage period on TL.

In conclusion, the lifestyle intervention with weight loss, increased physical activity, and healthy diet did not have an independent effect on TL since the TL was similar in the intervention and control groups during the 4.5 year follow-up period. However, TL may still play a role in the development of T2DM. Finally, our study provides strong evidence that leukocyte TL is a dynamic feature with the attrition rate correlating with the initial TL, and this is also seen in middle-aged, overweight or obese people with IGT.
